# Evaluation of Feature Selection for Alzheimer’s Disease Diagnosis

**DOI:** 10.3389/fnagi.2022.924113

**Published:** 2022-06-24

**Authors:** Feng Gu, Songhua Ma, Xiude Wang, Jian Zhao, Ying Yu, Xinjian Song

**Affiliations:** ^1^Department of Medical Image, Affiliated Nantong Rehabilitation Hospital of Nantong University, Nantong, China; ^2^Department of Medical Image, The Second People’s Hospital of Nantong, Nantong, China; ^3^Department of Neurology, Affiliated Nantong Rehabilitation Hospital of Nantong University, Nantong, China; ^4^Department of Neurology, The Second People’s Hospital of Nantong, Nantong, China; ^5^Nantong Center for Disease Control and Prevention, Nantong, China; ^6^Department of Rehabilitation Medicine, Affiliated Nantong Rehabilitation Hospital of Nantong University, Nantong, China; ^7^Department of Rehabilitation Medicine, The Second People’s Hospital of Nantong, Nantong, China

**Keywords:** artificial intelligence, Alzheimer’s disease, feature selection, stability, discriminability

## Abstract

Accurate recognition of patients with Alzheimer’s disease (AD) or mild cognitive impairment (MCI) is important for the subsequent treatment and rehabilitation. Recently, with the fast development of artificial intelligence (AI), AI-assisted diagnosis has been widely used. Feature selection as a key component is very important in AI-assisted diagnosis. So far, many feature selection methods have been developed. However, few studies consider the stability of a feature selection method. Therefore, in this study, we introduce a frequency-based criterion to evaluate the stability of feature selection and design a pipeline to select feature selection methods considering both stability and discriminability. There are two main contributions of this study: (1) It designs a bootstrap sampling-based workflow to simulate real-world scenario of feature selection. (2) It develops a decision graph to determine the optimal combination of supervised and unsupervised feature selection both considering feature stability and discriminability. Experimental results on the ADNI dataset have demonstrated the feasibility of our method.

## Introduction

Alzheimer’s disease (AD) (Xiao-Cong et al., 2018; Hou et al., 2020; Mishra and Li, 2020; Subasi, 2020; He et al., 2022) is a degenerative disease of the central nervous system, which is clinically manifested as progressive memory impairment, cognitive dysfunction, language dysfunction, and personality change, etc. AD has a serious impact on the lives of patients, but also brings a heavy economic burden to patients’ families. At present, the research progress of AD is slow, and the disease factors cannot be accurately determined. It is usually found at an advanced stage, and even treatment will not produce a better therapeutic effect. Therefore, the early diagnosis of AD is very critical, which can effectively inhibit the development of the disease, and even avoid the occurrence of clinical symptoms by taking timely treatment. Mild Cognitive Impairment (MCI) is considered as an intermediate state between health and AD. In patients with MCI, the probability of progressing to AD is about 10–15% (He et al., 2022). Therefore, if patients with MCI can be effectively identified and actively intervened, it is of great significance for the control of AD.

**FIGURE 1:**
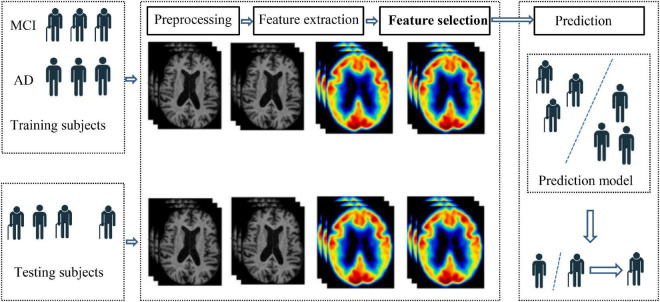
General process of MCI/AD recognition.

With the rapid development of artificial intelligence (Jiang et al., 2020; Xia et al., 2020; Zhang et al., 2020a,b, 2021a,b,^2022^), intelligent models are widely used in MCI or AD recognition. Kloppel et al. (2008) input gray matter features of brain images of AD patients into linear support vector machines (SVM), so as to apply the trained SVM to clinical studies. Ashburner and Friston (2000) applied morphometric methods to the diagnosis of AD, which spatially normalized high-resolution images of all subjects into the same stereotactic space. Then, gray matter was separated from the spatially normalized images and data smoothing was performed on them. Voxel parameter test statistics were performed on the two groups of smoothed gray images to improve the uneven intensity of the brain artifact images. Hinrichs et al. (2009) also proposed an AD recognition framework based on the smoothness of three-dimensional image coordinate space. It directly integrates the spatial relations of voxels into the learning framework and does not require image preprocessing information of other modes, thus automatically classifying subjects according to structural or functional imaging features. In addition, MCI was associated with changes in cortical morphology, such as cortical thickness, sulcus depth, surface area, gray matter volume, and mean curvature in different brain regions. These features have been shown to have a specific neuropathological and genetic basis. However, most methods have focused on univariate prediction models, and cortical features are usually isolated. Therefore, Li et al. (2014) used a multivariate approach to study the abnormalities of multiple cortical features in patients with mild cognitive impairment, and identified subtle patterns of changes in cortical anatomical structure through a classification model. Liu et al. (2013) used non-linear global data structure to map multivariable MRI data such as regional brain volume and cortical thickness into a low-dimensional local linear space through local linear embedding method, and trained a disease classifier by embedding brain features to predict whether MCI would be transformed into AD in the future. Möller et al. (2016) took the voxel values extracted from the voxel data as the original feature data, and proposed a feature selection method to apply to the original feature vector, so as to reduce the dimension of the original feature vector to a low-dimensional space and carry out the next classification task. From the above-mentioned studies, we can summarize the general process of MCI/AD recognition based on intelligent model, as shown in Figure 1. From Figure 1, it can be found that the general process of MCI/AD recognition contains four components, preprocessing, feature extraction, feature selection, and prediction. Preprocessing aims to process the original images including registration, standardizing and smoothing. Feature extraction aims to extract original features from the images after preprocessing. Feature selection aims to select discriminant features from the original feature set. Prediction aims to build a classification model to recognize MCI or AD patients. In the phase of prediction, based on the selected features, a prediction model is established for MCI/AD recognition.

From Figure 1, it can be found that feature selection is a key phase in the process of MCI/AD recognition. The goal of feature selection is to select discriminant features with low relevance between each other and high relevance to the outcome. In recent 2 years, some excellent feature selection work has emerged in the field of medical images. For example, Demir and Akbulut (2022) proposed a new residual- convolutional neural network to extract deep features from MRI images. Mainenti et al. (2022) proposed a radiomics-based pipeline to enhance MRI-based risk stratification in patients with endometrial cancer. Although previous studies have achieved great success in feature selection, feature discriminability is often the first important factor and feature stability is always omitted. In this study, first of all, feature stability, variance, and pairwise correlation were analyzed. Then, the least absolute shrinkage and selection operator (LASSO) and recursive feature elimination (RFE) were employed to search for the optimal feature set (Mainenti et al., 2022).

In this study, we focus on feature selection because few studies consider both the stability and performance of feature selection so far, which are two key factors for the classification phase. The main contributions cover two aspects. The first one is that we introduce a frequency-based criterion to evaluate the stability of a feature selection method. The second is that we propose a bootstrap-based flow chart and a decision graph to select the best combination of supervised and unsupervised feature selection methods. The following sections are organized as follows. Section “Data and Methods” presents the data we used and the methods we proposed. Section “Results” reports the experimental results, section “Discussion” discusses the experimental results and the last section concludes the whole study.

## Data and Methods

### Data

In this study, we select 103 patients with MRI and PET from the Alzheimer’s Disease Neuroimaging Initiative (ADNI) as our datasets. ADNI is a 5-year public partnership sponsored by several institutes, companies, and non-profit organizations (Zhang et al., 2021b). Owning to the original images cannot be directly used for our study, we set up a data preprocessing pipeline, which contains three main steps. Firstly, each subject in ADNI contains 96 PET images. Statistical parametric mapping (SPM) (Muzik et al., 2000) is used to fuse these PET images to construct a 3-D one which has brain spatial information and the feature information between tissue structures are also retained. In addition, motion correction is performed due to head motion. Secondly, the MRI image and PET image of each subject are registered, and affinely aligned. In the third step, the average template data generated is used to spatially normalize all PET images to the standard MNI space. PET images are also smoothed (8 mm Gaussian) to avoid the influences caused by noises. The AAL (automated anatomical atlas) (Rolls et al., 2020) which is available as a toolbox^1^ for SPM is used as a template to extract original features from PET images. Based on AAL, the brain is segmented into 116 regions, and we select 90 regions from the cerebrum for feature extraction. To be specific, firstly, the PET images are resampled to the same size as the AAL template so that each region is in correspondence spatially. The size of AAL template is 61 ×73 ×61. Then we extract average intensity values from all regions of PET images as original features for our proposed classification model.

### Methods

#### Stability Evaluation Metrics

In this study, we use a frequency-based criterion to measure the stability of a feature select method (Nogueira et al., 2017). For clarity, suppose we have a feature selection method Φ and a *d*-dimensional dataset *X*. The feature selection method is performed on the *d*-dimensional dataset *X* to select discriminant features. The feature selection process is repeated *M* times by a bootstrap strategy. Then we can define a binary matrix *Z*, as shown in (1) to indicate the feature selection results of *M* tries,


(1) $$Z=\left[\begin{array}{cccc} z_{11} & z_{12} & \ldots & z_{1\,d}\\ z_{21} & z_{22} & \ldots & z_{2\,d}\\ \ldots & \ldots & \ldots & \ldots \\ z_{M\,1} & z_{M\,2} & \ldots & z_{M\,d} \end{array}\right]$$

In *Z*, each row represents one try of feature selection. In each row, *z*_*ij*_ = 1(*i* = 1,2,…,*M*,*j* = 1,2,…,*d*) represents that the *j*-th feature is selected in the *i*-th try; otherwise, the *j*-th feature is not selected. Based on the binary matrix *Z*, the stability of feature selection method Φ in terms of the frequency-based criterion can be defined as:


(2) $$S\,t\,a\,b\,i\,l\,i\,t\,y\,\left(Z\right)=1-\frac{\frac{1}{d}\,\sum_{f=1}^{d}\left[\frac{M}{M-1}\,\left(\frac{1}{M}\,\sum_{i=1}^{M}z_{i\,f}\right)\,\left(1-\frac{1}{M}\,\sum_{i=1}^{M}z_{i\,f}\right)\right]}{\frac{\frac{1}{M}\,\sum_{i=1}^{M}\sum_{f=1}^{d}z_{i\,f}}{d}\,\left(1-\frac{\frac{1}{M}\,\sum_{i=1}^{M}\sum_{f=1}^{d}z_{i\,f}}{d}\right)}$$

From (2), we can see that *Stability*(*Z*) ranges from 0 to 1, the greater the value, the better the stability.

#### Stability Evaluation Workflow

**FIGURE 2:**
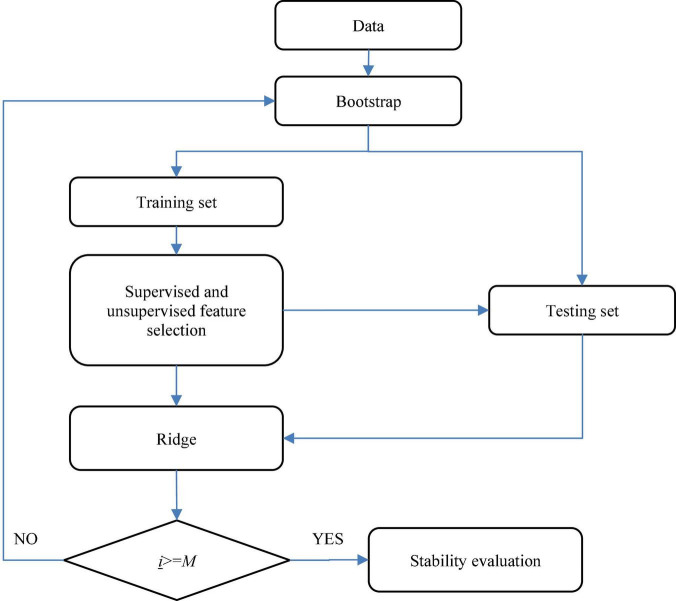
Flow chart for stability evaluation.

In this study, we use a supervised feature selection method to reduce features irrelative to the outcome, and an unsupervised feature selection method to reduce redundant features. To evaluate the stability of feature selection, a bootstrap sampling-based flow chart is established, which is shown in Figure 2. Firstly, the AD dataset is split into the training set (70%) and the testing set (30%) by bootstrap sampling. Then supervised and unsupervised feature selection is performed on the training set to select discriminant features. The testing set is updated with the selected features. Finally, a Ridge regression model is trained based on the selected features. The bootstrap sampling is repeated *M* times so that the matrix **Z** in (1) can be obtained. Based on **Z**, we can use (2) to evaluate the stability of the supervised and unsupervised feature selection methods we used.

#### Decision Graph for Feature Selection

In Li et al. (2017), a feature selection package was shared which contains 33 different kinds of supervised and unsupervised feature selection methods. In this study, we aim to choose a best supervised and unsupervised combination from this package for AD diagnosis. First of all, we set up an initial exclusion criterion to select a part of supervised and unsupervised feature select methods from the package provided by Li et al. (2017). The exclusion criterion states: (1) if prediction performance in terms of AUC of a feature selection method is lower than 0.5, the method is excluded. (2) If the running time of one try of a feature selection method is more than 30 min, the method is excluded. These exclusion criteria are defined for two reasons. The first is that if the prediction performance of the feature selection method is lower than 0.5, it indicates that the prediction performance of the method is close to the randomness level. Second, if the running time of a feature selection method exceeds 30 min, it will exceed the normal tolerance range when the training set size is not large. With the exclusion criterion, we finally select F score (denoted as S1:), T Score (denoted as S2), ReliefF (denoted as S3), and Fish Score (denoted as S4) as supervised feature selection methods, and Lap_score (denoted as U1), spectral feature selection (SPEC, denoted as U2), Monte Carlo feature selection (MCFS, denoted as U3), non-negative discriminative feature selection (NDFS, denoted as U4), unsupervised discriminative feature selection (UDFS, denoted as U5), and Person_score (denoted as U6) as unsupervised feature selection methods. Therefore, we have 24 combinations, i.e., S1U1, S1U2,…, S4U6, as shown in Table 1. Secondly, as we stated before that both performance and stability are important for Alzheimer’s disease diagnosis.

**TABLE 1:** All combinations of supervised and unsupervised feature selection methods.

Combination name	Name of supervised method	Name of unsupervised method
S1U1	F score	Lap_score
S1U2		SPEC
S1U3		MCFS
S1U4		NDFS
S1U5		UDFS
S1U6		Person score
S2U1	T score	Lap_score
S2U2		SPEC
S2U3		MCFS
S2U4		NDFS
S2U5		UDFS
S2U6		Person score
S3U1	ReliefF	Lap_score
S3U2		SPEC
S3U3		MCFS
S3U4		NDFS
S3U5		UDFS
S3U6		Person score
S4U1	Fish score	Lap_score
S4U2		SPEC
S4U3		MCFS
S4U4		NDFS
S4U5		UDFS
S4U6		Person score

**FIGURE 3:**
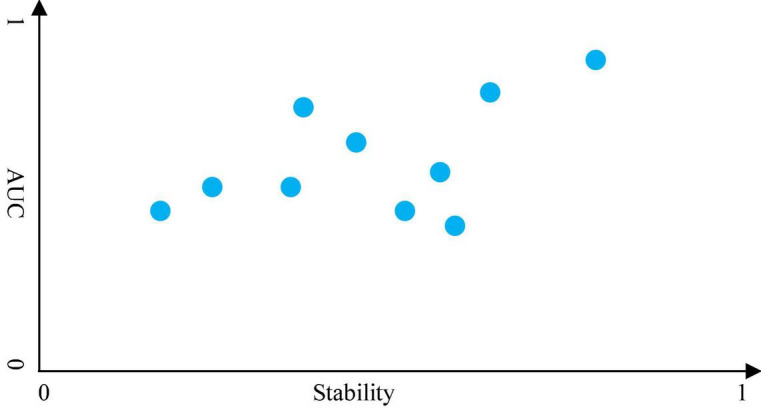
Decision graph for feature selection.

Based on Figure 2, we can generate the matrix **Z.** Thus, we can use (2) to evaluate the stability of the supervised and unsupervised feature selection methods we used. Therefore, we design a decision graph, as shown in Figure 3, to determine the best combination of the supervised and unsupervised feature selection methods.

## Results

**FIGURE 4:**
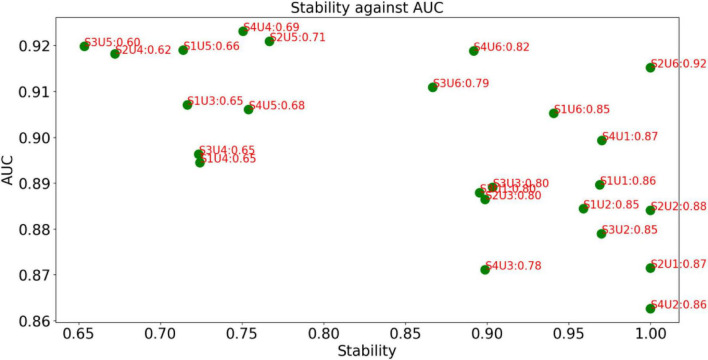
Decision graph for MRI features.

The decision graph of all combinations for MRI features is shown in Figure 4. It is observed that the combination S2U6 wins the best in terms of AUC*Stability, which means that the combination of T Score (supervised feature selection method) and Person Score (unsupervised feature selection method) performs better than other combinations in terms of both AUC and stability. Therefore, the supervised feature selection method *T Score* and the unsupervised feature selection method *Person Score* will be selected as the feature selection methods for modeling.

**FIGURE 5:**
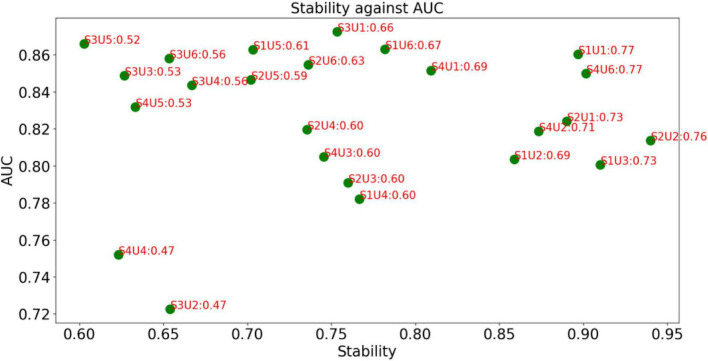
Decision graph for PET features.

The decision graph of all combinations for PET features is shown in Figure 5. Similar to Figure 4, it is observed that the combination S1U1 and S4U6 wins the best. Therefore, the combination *F score + Lap score* or the combination *Fish Score* + *Person Score* will be selected for the following phase of modeling.

From Figures 4, 5, it can be found that this is no combinations that always perform best. Our method is case-dependent, which means that it provides decision support for users.

## Discussion

In this study, we have 103 subjects, for both MRI and PET, the feature dimension of each subject is 93, which is near to the number of subjects. When classification models are applied to the high-dimensional data, a critical issue is known as the curse of dimensionality, which refers to the phenomenon that data becomes sparse in high-dimensional space may occur (Li et al., 2017). Therefore, feature selection plays a very significant role in the recognition of AD or MCI. So far, many feature selection methods have been successfully applied in the field of medical image-based diagnosis. For example, in Salvatore et al. (2015), employed PCA (principle component analysis) to select discriminant features from the density maps of WM (white matter) and GM (gray matter) as input of SVM for AD recognition. In Liu et al. (2013), employed LLE (local linear embedding) as the unsupervised feature reduction method to reduce features from the space of multivariate regional brain volume and cortical thickness MRI to a locally low-dimensional linear space while maintaining the global non-linear data structure. Then, the reduced brain features in the low-dimensional space were used to train the prediction model. Unlike Liu et al. (2013) and Salvatore et al. (2015) in Beheshti et al. (2015) proposed a filter-based supervised feature reduction method containing three main steps. First of all, feature extraction was carried out by using the voxel clusters that are detected by the voxel-based morphometric (VBM) on sMRI and the voxel values as the volume of interest (VOI). Secondly, the probability distribution function of the VOI was employed to represent the statistical information of the respective high-dimensional structural MRI samples. Thirdly, the final selected features were employed to train a SVM classifier to perform the AD recognition task. In Nir et al. (2015) extracted DTI-based features and proposed a tractography-based model to recognize AD and MCI. First of all, the authors used tractography and clustering techniques to locate and organize fibers into 18 fiber bundles. Secondly, the authors computed density maps to quantify the number of fibers passing through each voxel and used the shortest path graph search to reduce the fiber bundles based on maximum density path (MDP) so that the fiber bundles can be expressed in a compact and low-dimensional space. Thirdly, the diffusivity measures of fractional anisotropy (FA) and MD computed along all the registered across subjects (MDPs) were selected as the features to train an SVM classifier. Feature selection methods in this category can be characterized as making use of the global or local statistical information. In De Martino et al. (2008) employed multivariate feature selection to select features to model functional MRI spatial patterns. To be specific, the authors employed RFE combined with an SVM classifier (REF-SVM) to reduce the irrelevant voxels recursively. Similarly, in Wee et al. (2011), based on DTI images, Wee et al. proposed a framework for MCI recognition. In this framework, the original features come from the anatomical regions, and REF-SVM was also used to reduce the original feature set.

Although different kinds of feature selection (reduction) methods have been widely used for AD and MCI recognition, an important thing that is not fully considered is the stability of the feature selection methods. In practice, we expect that the selected feature selection method can maintain robustness when training data changes slightly. Therefore, in this study, we introduce a frequency-based criterion to evaluate the stability and design a pipeline to select feature selection methods considering both stability and discriminability. Experimental results shown in Figures 4, 5 indicate that the proposed pipeline works well and can help us to determine the best combination of feature selection methods. That is to say, the proposed criterion AUC*Stability can find the optimal combination of supervised and unsupervised feature selection methods.

## Conclusion

In this study, we introduce a frequency-based criterion to evaluate the stability of feature selection and design a pipeline to select feature selection methods considering both stability and discriminability.

## Data Availability Statement

Publicly available datasets were analyzed in this study. The data is available on http://adni.loni.usc.edu/about/.

## Author Contributions

FG and SM contributed to the writing and experiments. XW, JZ, and YY contributed to the data collection and preprocessing. XS supervised the study. All authors contributed to the article and approved the submitted version.

## Conflict of Interest

The authors declare that the research was conducted in the absence of any commercial or financial relationships that could be construed as a potential conflict of interest.

## Publisher’s Note

All claims expressed in this article are solely those of the authors and do not necessarily represent those of their affiliated organizations, or those of the publisher, the editors and the reviewers. Any product that may be evaluated in this article, or claim that may be made by its manufacturer, is not guaranteed or endorsed by the publisher.

## Footnotes

1
http://www.gin.cnrs.fr/AAL
